# Measuring the quality of interprofessional collaboration in child mental health collaborative care

**Published:** 2012-02-10

**Authors:** Cécile Rousseau, Audrey Laurin-Lamothe, Lucie Nadeau, Suzanne Deshaies, Toby Measham

**Affiliations:** McGill University, CSSS de la Montagne (CLSC Park Extension), Youth Mental Health, 7085 Hutchison Street, Local 204.2, Montreal, Quebec, Canada, H3N 1Y9; McGill University, CSSS de la Montagne (CLSC Park Extension), Youth Mental Health, 7085 Hutchison Street, Local 204.2, Montreal, Quebec, Canada, H3N 1Y9; McGill University, CSSS de la Montagne (CLSC Park Extension), Youth Mental Health, 7085 Hutchison Street, Local 204.1, Montreal, Quebec, Canada, H3N 1Y9; Community Psychologist, CSSS de Bordeaux-Cartierville Research Center—Saint-Laurent, University Affiliated Center, 11 822 du Bois-de-Boulogne Boulevard, Montreal, H3M 2X6; McGill University, CSSS de la Montagne (CLSC Park Extension), Youth Mental Health, 7085 Hutchison Street, Local 204.1, Montreal, Quebec, Canada, H3N 1Y9

**Keywords:** interprofessional, children, mental health, collaborative care, collaboration, instruments

## Abstract

**Objective:**

This pilot study examines the potential utility of the Perception of Interprofessional Collaboration Model and the shared decision-making scales in evaluating the quality of partnership in child mental health collaborative care.

**Methods:**

Ninety-six primary care professionals working with children and youth responded to an internet survey which included the Perception of Interprofessional Collaboration Model scale (PINCOM-Q) and an adapted version of a shared decision-making scale (Échelle de confort décisionnel, partenaire—ECD-P). The perceptions of child mental health professionals were compared with those of other professionals working with children.

**Results:**

The PINCOM-Q and the ECD-P scales had an excellent internal consistency and they were moderately correlated. Child mental health professionals’ Individual Interprofessional Collaboration scores from the PINCOM-Q individual aspects subscale were better than that of other child professionals.

**Conclusion:**

These scales may be interesting instruments to measure the quality of partnership in child mental health collaborative care settings. Research needs to replicate these findings and to determine whether the quality of collaboration is a predictor of mental health outcome.

## Introduction

In order to address the needs for mental health care, primary health care delivery has been undergoing reform in a number of countries such as Canada, Brazil, and the UK [1]. In some places, such as in the UK, primary care settings have been promoted as a major site of delivery of mental health services [2]. In Quebec, Canada, a primary care reform was enacted in 2005 which prioritizes primary care settings as the main site of mental health care delivery for both adults and youth [3]. The pressing need for services for youth, as evidenced, for example, by Quebec ranking among the highest rates of suicide in the world, the concerns that youth needs were not being adequately addressed, and the acknowledgement that most youth could be treated in less specialized care settings [3] were among some of the reasons identified for the major youth mental health services reform. Collaborative care models, also called shared care, have the objective of strengthening and supporting primary care professionals to take over the youth mental health mandate. They are based on a strong partnership between first line health and social service care providers and specialized mental health resources [4, 5].

This has meant forging collaborations among a vast system of service providers, as child mental health is often delivered through a number of practitioners, institutions and systems. For example, mental health is provided in schools by school psychologists and psycho educators, by practitioners in juvenile justice systems for youth with legal difficulties, by child welfare system professionals for youth in danger of abuse or neglect; by hospital departments through specialists in mental health such as psychologists, psychiatrists and social workers, by primary care physicians, social workers and psycho educators, and by a number of other therapists practicing in institutions and in the community. In Quebec, prior to the reform, there were a number of formal and informal systems that linked practitioners, institutions and networks together. The reform has introduced a new system organising collaborations and partnerships. It requires building collaboration and partnerships between persons, institutions, and systems which may or may not have experiences of working together in the past.

Although shared care models are promising, there are still relatively few studies examining their outcomes in adult mental health [6, 7] and even fewer in child mental health [8–10]. Many studies suggest that the quality of the collaboration among partners is the key element in the success of collaborative care models [11]. In these settings collaboration is defined as an active and on-going partnership between professionals and institutions with diverse backgrounds and mandates, who work together to provide services [12]. Thus, being able to measure collaboration effectively will provide an important contribution to evaluating the efficacy of shared care models, and this in turn has implications for mental health care practice for youth and for further policy decisions aimed at improving youth mental health care practice. Currently one of the main methodological obstacles in evaluating collaborative care models is the lack of tools to assess the quality of collaboration.

### Measuring key aspects of collaboration and partnerships

The establishment of a trusting and respectful relationship is an important factor in interprofessional collaboration [13]. Professionals need to know each other and to reach a shared understanding of their expertise and roles [14, 15]. A positive attitude towards collaboration needs to be promoted [16] but is not in itself sufficient. Specific opportunities for interprofessional communication are also needed to exchange ideas and debate mandates and clinical perspectives [17]. Finally, the cultures of the relevant institutions need to deliberately facilitate collaborative practices in order to support new patterns of interprofessional relations [18].

A review of the determinants of successful collaboration identifies three groups of factors: the process related to interpersonal and interprofessional relationships within the team, the organizational conditions and the specificities of the wider environment surrounding the organization. The review underlines the relative lack of empirical studies on these determinants and argues in favour of a systemic approach to evaluative research in this field. While adopting a different perspective, a theoretical model of measurement of integrated care also distinguishes horizontal integration from intra-organizational and inter-organizational integration, emphasizing the need for a systemic evaluation of this dimension [19].

Kodner and Sprevenberg [20] recognize that measuring integration and collaboration is a very complex task and argue that these assessments need to be put in relation to not only a wide range of patient outcomes but also to the subjective perceptions of the patients themselves. There are currently very few instruments to operationalize these concepts in collaborative mental health care, and the majority of instruments lack sufficient psychometric and theoretical development (for a review of current instruments measuring Interprofessional Collaboration and interprofessional education please see Thannhauser et al. [21]).

One promising scale in youth mental health is the Perception of Interprofessional Collaboration Model questionnaire (PINCOM-Q) constructed by Ødegård [22, 23]. This instrument was built on the basis of a literature search documenting the different key dimensions of collaboration in child mental health care, and measures different aspects of interprofessional collaboration in child mental health care. It was piloted in Western Norway and appeared to be a promising instrument which may be able to help improve interprofessional collaboration by indicating ways to enhance interprofessional dialogue and by providing a way to monitor changes in perceptions of interprofessional collaboration over time [24].

The concept of shared decision-making, which initially promoted the empowerment of patients and natural caregivers, is another useful concept to appraise the involvement of all persons around a patient and their participation in health care decisions [25]. It describes the process by which a practitioner and a patient reach an agreement about health care choices, and is considered to be the crux of patient-centered care [26]. In the context of child collaborative mental health care the process of shared decision-making involves not only the family but also the whole team of professionals involved. Assessing shared decision-making in this setting provides a window on the quality of the collaboration in the inter-professional team around a specific clinical situation. It constitutes an evaluation of professional behaviours and actions which is complementary to an inquiry about professional perceptions of the interprofessional collaboration. Different scales have been proposed to assess decisional processes. The ‘Decisional Conflict Scale’ (DCS) [27] and the ‘Satisfaction with Decision Scale’ have been adapted and validated in French under the name ‘Échelle de confort décisionnel du médecin’ (ECD-M) [25], which measures the level of comfort of the care provider with a particular clinical decision.

### Objective

The aim of this pilot study was to determine the potential usefulness of two scales, PINCOM-Q) [22] and the ECD-P (Échelle de confort décisionnel—partenaire) which is an adaptation of the ECD-M, in order to evaluate the quality of partnership in child mental health collaborative care. The specific objectives were [1] to establish the psychometric characteristics of these instruments in this specific setting; to study the association between the two scales; and to compare the scores of the two instruments rated by different teams involved in collaborative care in child mental health.

## Method

### Setting: the Quebec mental health plan

In 2004, 95 integrated health networks were established in the province, called Local Service Networks (RLS: Réseaux locaux de services). Each of these networks included a Health and Social Service Centre (CSSS: Centre de Santé et des services sociaux), which merged local primary care community service centres (CLSCs: Centre local de services communautaires), and sometimes, local hospitals. The RLS included partnerships with other hospitals (including university hospitals) and other institutions like schools and youth protection centres. These new networks received the mandate of managing the health and social service needs of their designated population bases. In youth mental health, the institutions within each network were to form partnerships together, bringing together schools, youth centres, community family physicians, community psychologists, universities and other partners, in order to ensure that youth in their territories received mental health care. It was envisaged that with this new plan the majority of mental health care needs would be delivered by the primary care services and their community partners. In order to achieve these goals, the primary care teams of the CSSS centres, which already had teams dedicated to the general psychosocial care of children and to improving students’ health and well being by collaborating with schools, were charged with developing youth mental health teams in order to support the primary care professionals, including their CSSS general psychosocial care and school teams, in delivering youth mental health care in their area. To help accomplish this, each CSSS would have an intake triage system (where youth referred by primary care services would be evaluated and oriented towards a hierarchy of mental health services both within and external to the CSSS), a newly created youth mental health team to provide care and to support other primary care teams, and a consultant psychiatrist designated by various hospitals’ psychiatric departments to provide advice and support to the CSSS primary care service network.

### Sample and procedure

Clinicians from the child and youth teams in three CSSSs serving multi-ethnic neighbourhoods’ in Montreal (Quebec, Canada), were invited to participate in an internet survey (Lime survey) about their experience of partnership in their work. The targeted participants included the CSSSs multidisciplinary professionals of the newly created youth mental health teams, as well as the professionals in the already established general child psychosocial care teams and the school collaboration teams. These teams were all located within the CSSS’s CLSCs. This on-line survey was proposed to the 165 professionals working within the youth mental health, general psychosocial care and school teams of the three CSSSs.

### Instruments

The survey included PINCOM-Q and the ECD-P, as well as questions on obstacles to partnership, facilitating factors, and sociodemographic variables. The PINCOM-Q addresses the general perceptions of clinicians about collaborations while the ECD-P focuses on the appraisal of a specific clinical interaction, documenting perceptions about collaboration involving an actual clinical event.

The PINCOM-Q [22] has been designed to address the interdisciplinary and the interinstitutional context which characterizes child mental health collaborative care. This scale documents the perceptions and behaviours of clinicians around collaboration. It is composed of three dimensions assessing individual, group and organizational collaborations. Individual aspects of collaboration include professional power, role expectations, personality style and work motivation. Group aspects of collaboration describe leadership, coping abilities, communication and social support. Finally, organizational aspects of collaboration document organizational aims, environment, culture and domain. The scale is composed of 48 items rated on a 7 degree Likert scale.

The ECD-P is an adaptation of the ECD-M which has been validated in French. The ECD-M instrument measures different partners’ levels of comfort with a particular clinical decision. It is composed of 16 items rated on a 5Ŷ Likert scale and has good psychometric properties. Based on previous qualitative research in collaborative care [28] we added 3 items to the ECD-M specifically targeting the impact of interinstitutional relations in the clinical decision-making process. We called this 19 item instrument, the ECD-P.

### Statistical analyses

The following tests were done: 1) a descriptive analysis of the professionals’ sociodemographic profiles (mean, standard deviation and range); 2) Cronbach's alpha for the two scales and for the PINCOM-Q subscales; 3) bivariate and multivariate analysis (T-test and ANOVA) of the two scales scores for the different mental health (n=19), school (n=17) and general psychosocial (n=18) teams.

The study received approval from the Ethical Review Board of the CSSS de la Montagne.

## Results

### Descriptive analysis of the participants’ sociodemographic profiles

Of the 165 professionals invited to take part in the online survey, 103 (62.42%) visited the survey site, and 96 (58.18%) responded to the questionnaire, although they did not always complete it, which resulted in some missing data.

Participants were social workers, psycho educators, psychologists, art therapists, educators, nurses and consulting child psychiatrists. Most respondents were women (n=56, 91.8%) [See Table 1].

Only five respondents (8.5%) were 20–29 years old, 33.9% (n=20) were between 30 and 39 years old, another third were between 40 and 49 years old, and fourteen participants (23.7%) were more than 50 years old. French was the mother tongue of 69.1% (n=38) of the sample and English the mother tongue of 30.9% (n=17) of the participants. All respondents reported being able to express themselves in English and French. Sixty-eight per cent (n=42) of the professionals were born in Québec.

### The psychometric characteristics of the instruments

Internal consistency estimates of reliability computed for the PINCOM-Q and the ECD scales gave values for Cronbach Alpha of 0.90 for the original ECD-M scale, and of 0.93 for the ECD-P scale which included the three added items. The PINCOM-Q Cronbach’s alpha was 0.94. The Cronbach’s alpha for the PINCOM-Q subscales are presented in Table 2.

### Associations within and between the PINCOM-Q and the ECD-P

The Pearson Correlation between the Individual Collaboration subscale of the PINCOM-Q and the Group Collaboration and Organizational Collaboration subscales were respectively R=0.626, p<0.0001, and R=0.388, p<0.0001. The correlation between the Group and the Organizational Subscales was R=0.732, p<0.0001, indicating that these dimensions are strongly associated. For the overall sample, the Pearson correlation between the ECD-M scale and the PINCOM-Q global scores was statistically significant (R=0.356, p=0.003). The Pearson correlation between the ECD-P and the PINCOM-Q was (R=0.411, p<0.0001).

### The scores of the two scales according to care team

The comparison of the different teams’ PINCOM-Q scores and PINCOM-Q sub-scores and the ECD-P scale score indicated that child mental health team professionals reported more positive perceptions of interprofessional collaboration and more comfort in shared decision-making than the professionals of the other teams, although in most cases the differences were not statistically significant.

The PINCOM-Q subscale scores measuring individual perceptions and behaviours around interprofessional collaborations were significantly better for the child mental health professionals

## Discussion

The Perception of Interprofessional Collaboration Model scale (PINCOM-Q) and the modified shared decision-making scale (ECD-P) both showed good internal consistency when used in this collaborative care setting. As theoretically expected, the fact that they are moderately positively correlated indicates that the level of comfort with a clinical decision in a partnership setting is associated with the perception of interprofessional relations. This moderate correlation also underlines that the two scales are measuring different dimensions of partnership, and that it would be useful to use them both in a complementary way when assessing the quality of collaborations. A recent theoretical model [29] has emphasized the value of merging perceptions of interprofessional relations and the comfort with decision-making to appraise partnerships, but there has been no operationalization of such a model in child mental health.

This study also found that the child mental health professionals had better perceptions of collaboration and more comfort with clinical decisions in the collaborative care context than the other youth teams. This only reached significance in the case of individual aspects of collaboration. These results confirm qualitative research findings describing the same institutional context, which have highlighted that the strong emphasis put on interprofessional collaboration in the newly created youth mental health teams have created cohesive teams with highly motivated professionals [28]. This finding may also reflect the greater tensions experienced by primary care health professionals working in the school environment—because of the divergent institutional mandates—the schools’ aim is first and foremost pedagogical, while the health professionals’ mandate is promoting health and wellbeing. Overall, these results also suggest that the PINCOM-Q and the ECD-P scales may be quite sensitive to variations in the quality of collaboration. As such, they may constitute interesting indicators of the quality of the partnership in child mental health collaborative care settings.

In complex domains like collaborative care, developing and implementing research is a challenge because of the multiplicity of variables involved in systemic interventions [30]. Although qualitative research is absolutely key to document the process of implementation and to identify the main factors associated with successful models [31, 32], the absence of quantitative indicators to monitor collaborative care models may be one of the factors contributing to the relative underrepresentation of research on these services. As research can be a strong lever for transforming services [33] and assessing outcome, it is essential to develop tools like the PINCOM-Q and the ECD-P to monitor and assess the development of collaborative care programs. The small sample size and the relatively low response rate are, however, important limitations of this pilot study. A qualitative appraisal of the quality of partnership would further expand our understanding of the usefulness of the scale to assess a successful partnership.

## Conclusion

Measuring the quality of interprofessional and interinstitutional collaboration is a challenge in the evaluation of collaborative care settings. Although the results of the present pilot study are very preliminary, they suggest that the PINCOM-Q and the ECD-P may be promising scales to measure different dimensions of the quality of collaboration in collaborative care settings. A factorial analysis would be needed to investigate their possible complementarity further. Possible implications for practice and services include using these scales to monitor the implementation of new collaborative care models. The scales could also be used, before and after training in collaborative care, to assess to what extent the training has altered the practices of professionals and increased their comfort with shared decision-making. Internationally, this could support transnational comparison of very different mental-health care services which are organized around the principle of interprofessional collaboration. Future studies need to establish if the quality of collaboration is in fact associated with improved outcomes for children and families seeking care. Beyond improving intervention, a successful collaborative network may be especially important to reach out to and prevent the social exclusion of vulnerable children as these children are known to underutilize mental health services [34].

## Figures and Tables

**Table 1.** Profile of participating professionals.

	n	%
Gender		
Female	56	91.8
Male	5	8.2
Age		
20–29 years old	5	8.5
30–39 years old	20	33.9
40–49 years old	20	33.9
50–59 years old	12	20.3
60 years old and over	2	3.4
Mother tongue		
French	38	69.1
English	17	30.9
Other	55	100
Fluency in English		
Yes	60	100
Fluency in French		
Yes	61	100
Born in Quebec		
Yes	42	68.9
No	19	31.1
Father born in Quebec		
Yes	36	61
No	23	39
Mother born in Quebec		
Yes	36	59
No	25	41
Experience in their occupation		
1–5 years	9	15.3
6–10 years	11	18.6
11–20 years	21	35.6
More than 20 years	18	30.5
Experience in the primary care institution		
Less than one year	4	6.8
1–5 years	20	33.9
6–10 years	15	25.4
11–20 years	16	27.1
More than 20 years	4	6.8
Team		
Mental health team	19	35.2
School team	17	31.5
General child psychosocial	18	33.3
Experience with the team		
0–6 months	2	3.4
6–12 months	7	11.9
1–3 years	24	40.7
3–5 years	13	22
More than 5 years	13	22

**Table 2.** Comparison between the ECD-M Scale, ECD-P Scale and Perception of Interprofessional Collaboration scores PINCOM-Q of the primary care child teams.

	Mental health team		School team		Other		ANOVA (F)	p	Cronbach's alpha
Scale	Mean	Standard deviation	Mean	Standard deviation	Mean	Standard deviation			
PINCOM.Q—total	136.21	33.64	159.29	32.55	147.89	27.93	2.41	0.1	0.973
(48 items)									
PINCOM.Q—individual	49.11	14.15	60.06	13.21	57.61	11.06	3.64	0.03	0.793
(16 items)									
PINCOM.Q—group	40.32	13.96	50	15.24	43.33	10.47	2.44	0.1	0.907
(16 items)									
PINCOM.Q—organization	46.79	10.31	49.24	8.65	46.94	12.26	0.3	0.76	0.864
(16 items)									
ECD-M	26.53	10.78	30.29	6.79	27.61	9.65	0.77	0.47	0.902
(16 items)									
ECD-P	37.21	15.03	43.12	8.51	39.72	14.6	0.91	0.41	0.931
(19 items)									

*A lower mean indicates a more positive perception of collaboration or more comfort with shared decision-making. PINCOM-Q=Perception of Interprofessional Collaboration. ECD-P=Shared decision-making scale – partner.

## References

[1] Goldman J, Meuser J, Lawrie L, Rogers J, Reeves S (2010). Interprofessional primary care protocols: a strategy to promote an evidence-based approach to teamwork and the delivery of care. Journal of Interprofessional Care.

[2] Carpenter J, Barnes D, Dickinson C, Wooff D (2006). Outcomes of interprofessional education for community mental health services in England: the longitudinal evaluation of a postgraduate programme. Journal of Interprofessional Care.

[3] Ministère de la santé et des Services sociaux (2005). Plan d'action en santé mentale 2005–2010: La force des liens.

[4] Chenven M (2010). Community systems of care for children's mental health. Child Adolescent Psychiatric Clinic of North America.

[5] Cockburn K, Bernard P (2004). Child and adolescent mental health within primary care: A study of general practitioners' perceptions. Child and Adolescent Mental Health.

[6] Mayer E, Kirmayer  LJ, Lemelson R, Barad M (2007). Somatic manifestations of traumatic stress. Understanding trauma: biological, clinical and cultural perspectives.

[7] Kates N, Ackerman S (2002). Shared mental health care in Canada: a compendium of current projects.

[8] Richardson L, McCauley E, Katon W (2009). Collaborative care for adolescent depression: a pilot study. General Hospital Psychiatry.

[9] Abrahams S, Udwin O (2002). An evaluation of a primary care-based child clinical psychology service. Child and Adolescent Mental Health.

[10] Puura K, Hilton D, Papadopoulou K, Tsiantis J, Ispanovic-Radojkovic V, Rudic N (2002). The European early promotion project: a new primary health care service to promote children's mental health. Infant Mental Health Journal.

[11] Craven MA, Bland R (2006). Meilleures pratiques pour des soins de santé mentale axés sur la collaboration: Une analyse des données existantes. Revue canadienne de psychiatrie.

[12] Barr H, Koppel I, Reeves S, Hammick M, Freeth D (2005). Effective interprofessional education. Argument, assumption and evidence.

[13] Gerardi D, Fontaine DK (2007). True collaboration: envisioning new ways of working together. AACN Advanced Critical Care.

[14] Gask L (2005). Overt and covert barriers to the integration of primary and specialist mental health care. Social Science and Medicine.

[15] Railton S, Mowat H, Bain J (2000). Optimizing the care of patients with depression in primary care: the views of general practitioners. Health and Social Care in the Community.

[16] Byng R, Jones R (2004). Mental health link: the development and formative evaluation of a complex intervention to improve shared care for patients with long-term mental illness. Journal of Evaluation in Clinical Practice.

[17] Rockman P, Salach L, Gotlib D, Cord M, Turner T (2004). Shared mental health care. Model for supporting and mentoring family physicians. Canadian Family Physician Le Médecin de famille canadien 2004 March.

[18] San Martín-Rodríguez L, Beaulieu M-D, D'Amour D, Ferrada-Videla M (2005). The determinants of successful collaboration: a review of theoretical and empirical studies. Journal of Interprofessional Care.

[19] Ahgren B, Axelsson R (2005). Evaluating integrated health care: a model for measurement. International Journal of Integrated Care [serial online].

[20] Kodner DL, Spreeuwenberg C (2002). Integrated care: meaning, logic, applications, and implications—a discussion paper. International Journal of Integrated Care [serial online].

[21] Thannhauser J, Russell-Mayhew S, Scott C (2010). Measures of interprofessional education and collaboration. Journal of Interprofessional Care.

[22] Ødegård A (2006). Exploring perceptions of interprofessional collaboration in child mental health care. International Journal of Integrated Care [serial online].

[23] Ødegård A (2007). Time used on interprofessional collaboration in child mental health care. Journal of Interprofessional Care.

[24] Ødegård A, Strype J (2009). Perceptions of interprofessional collaboration within child mental health care in Norway. Journal of Interprofessional Care.

[25] Légaré F, Graham ID, O'Connor AM, Dolan JG, Bélanger-Ducharme F (2003). Prise de décision partagée: Traduction et validation d'une échelle de confort décisionnel du médecin. Pédagogie Médicale.

[26] Weston WW (2001). Informed and shared decision-making: the crux of patient centered care. Canadian Medical Association Journal.

[27] O’Connor AM (1995). Validation of a decisional conflict scale. Medical decision-making.

[28] Légaré F, Stacey D, Pouliot S, Gauvin F-P, Desroches S, Kryworuchko J (2011). Interprofessionalism and shared decision-making in primary care: a stepwise approach towards a new model. Journal of Interprofessional Care.

[29] Nadeau L, Rousseau C, Seguin Y, Moreau N (2009). Évaluation préliminaire d'un projet de soins concertés en santé mentale jeunesse à Montréal: faire face à l'incertitude institutionnelle et culturelle. Santé mentale au Québec.

[30] Frost N, Lloyd A (2006). Implementing multi-disciplinary teamwork in the new child welfare policy environment. Journal of Integrated Care.

[31] Naar-King S, Siegel PT (2000). A model for evaluating collaborative health care programs for children with special needs children's services. Social Policy, Research and Practice.

[32] Salmon G, Rapport F (2005). Multi-agency voices: a thematic analysis of multi-agency working practices within the setting of a child and adolescent mental health service. Journal of Interprofessional Care.

[33] Jarrett D, Stevenson T, Huby G, Stewart A (2009). Developing and implementing research as a lever for integration: The impact of service context. Journal of Integrated Care.

[34] Edwards A (2004). The new multi-agency working: collaborating to prevent the social exclusion of children and families. Journal of Integrated Care.

